# Biliary atresia: the role of gut microbiome, and microbial metabolites

**DOI:** 10.3389/fcimb.2024.1411843

**Published:** 2024-07-22

**Authors:** Sansan Feng, Yongkang Cheng, Chuqiao Sheng, Chunfeng Yang, Yumei Li

**Affiliations:** Department of pediatric intensive care unit, Children’s Medical Center, The First Hospital of Jilin University, Changchun, China

**Keywords:** biliary atresia, gut microbiota, metabolites, probiotics, children

## Abstract

Biliary atresia (BA) is a progressive fibroinflammatory disease affecting both the extrahepatic and intrahepatic bile ducts, potentially leading to chronic cholestasis and biliary cirrhosis. Despite its prevalence, the exact mechanisms behind BA development remain incompletely understood. Recent research suggests that the gut microbiota and its metabolites may play significant roles in BA development. This paper offers a comprehensive review of the changing characteristics of gut microbiota and their metabolites at different stages of BA in children. It discusses their influence on the host’s inflammatory response, immune system, and bile acid metabolism. The review also explores the potential of gut microbiota and metabolites as a therapeutic target for BA, with interventions like butyrate and gut microbiota preparations showing promise in alleviating BA symptoms. While progress has been made, further research is necessary to untangle the complex interactions between gut microbiota and BA, paving the way for more effective prevention and treatment strategies for this challenging condition.

## Introduction

Biliary atresia (BA) is a severe hepatobiliary disease of unknown etiology and pathogenesis in infancy. It is characterized by varying degrees of fibrotic atresia of the extrahepatic and intrahepatic bile ducts, resulting in obstructive jaundice and liver fibrosis (Hartley et al., 2009). BA is a rare disease with incidence rates ranging from 1 in 18,000 live births in European and American countries to 1 in 8,000 in Asian countries. Females are 3.5-4.0 times more likely to be affected than males (Sanchez-Valle et al., 2017; Schreiber et al., 2022). The current treatment of choice for BA is Kasai hepatoportoenterostomy (KPE), which is successful in about 50% of patients (Hartley et al., 2009). Despite advancements in surgical techniques for BA, the clinical outcomes for these patients remain unsatisfactory. Recurrent cholangitis and progressive liver fibrosis often occur within a short time period after KPE, necessitating liver transplantation (LT). Therefore, a clear understanding of the etiology and molecular pathogenesis of BA is crucial for gaining insight into disease progression and identifying potential intervention strategies.

The term “gut microbiota” encapsulates the intricate and diverse assembly of microorganisms, including bacteria, fungi, archaea, and viruses, that reside within the gastrointestinal tract. The resident gut microbiota and its host engage in a symbiotic relationship, wherein each party derives significant benefits from the other. The gut microbiota, in a state of mutual dependence, flourishes within the stable and nutrient-rich environment furnished by the host. In turn, it generates bioactive metabolites that are pivotal for sustaining intestinal homeostasis and bolstering the host’s overall health (Valdes et al., 2018; Jaswal et al., 2023). It also influences the physiological functioning of distant organs through several routes, including liver, endocrine, immune, and metabolic pathways. This is particularly well documented regarding the gut-liver axis (Delzenne et al., 2019; Tilg et al., 2022; Rager and Zeng, 2023). Typically, the composition and structure of gut microbiota and metabolites are relatively balanced, and they communicate bi-directionally with the gut and its microbiome through the gut-liver axis (Milosevic et al., 2019; Tilg et al., 2022; Rager and Zeng, 2023). Breaking this balance, termed dysbiosis, can trigger an excessive intestinal immune response and metabolic disorders, which leads to the disease (Milosevic et al., 2019; Hou et al., 2022). Recent research has suggested that the gut microbiota and their metabolites, the diverse community of microorganisms residing in the gastrointestinal tract, may play a crucial role in the pathogenesis of hepatobiliary diseases, including nonalcoholic fatty liver disease, as well as liver fibrosis, cholestatic liver disease and BA (Kobayashi et al., 1988; Aron-Wisnewsky et al., 2020; Schneider et al., 2021). Understanding the relationship between the gut microbiota and their metabolites and hepatobiliary diseases shows great potential in treating liver disease.

Growing evidence suggests that gut microbiota and metabolites are associated with BA (Song et al., 2021b; Yang et al., 2022). A few cohort studies with few subjects have found that individuals with BA have different gut bacterial communities from healthy individuals (Han, 2019; Chen, 2022). Moreover, A 2-year randomized, double-blind, placebo-controlled trial found that supplementing *Lactobacillus casei rhamnosus* (LGG) significantly reduced frequency of bacterial cholangitis in 30 patients with BA (Orłowska et al., 2021). However, the systemic and functional link between gut microbiota and their metabolites, and BA has remained unexplored. Therefore, this review summarizes the possible etiology of BA, the correlation between the gut microbiota and its metabolites and BA, the possible mechanisms for BA, and the causal effect of gut microbiota on BA. We also discuss the importance of gut microbiota and metabolites (i.e., probiotics, butyrate) as a plausible therapeutic administration strategy.

## Etiology of biliary atresia

**Figure 1:**
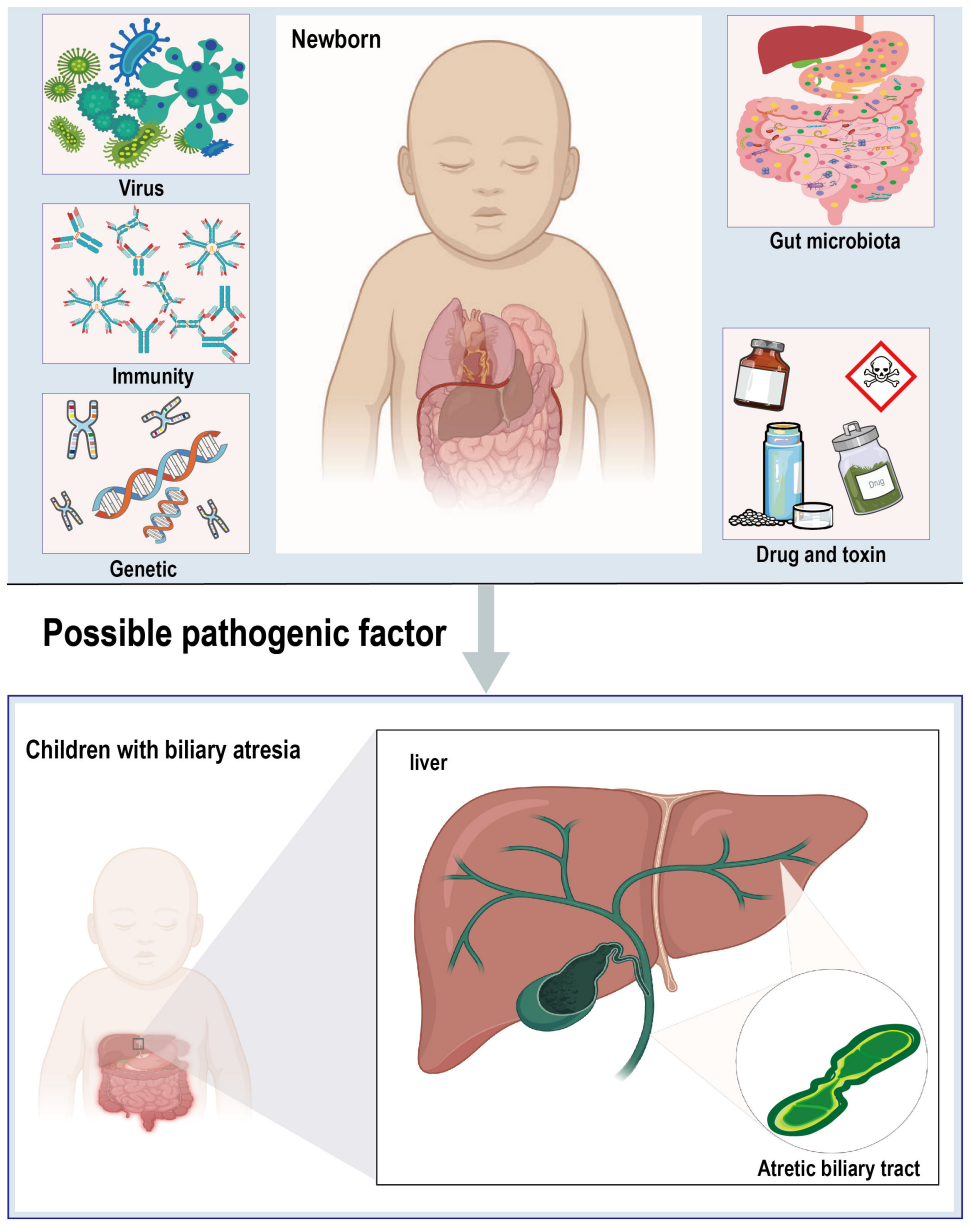
Diagrammatic representation of biliary atresia and multi-factorial etiology.

The pathogenesis of BA is highly complex, with the precise etiology and pathogenesis still not fully understood. Current research suggests that a multifactorial origin is likely, with viral infections, toxins, genetic variants, and immune dysregulation being the most commonly implicated factors in the development of the disease (Hartley et al., 2009; Bezerra et al., 2018). Additionally, imbalances in the gut microbiota and its metabolites are considered as potential contributors to the pathogenesis of BA (**Figure 1**).

### Viral infection

Viral infections have been postulated to play a role in the etiology of the disease (Averbukh and Wu, 2018). However, the mechanism through which virus infection causes these severe abnormalities during human pregnancy is not clear (Averbukh and Wu, 2018). In the 1970s, Altshuler first suggested finding evidence of an association between BA and viral perinatal infections (Altshuler, 1973). Several studies have detected a notably high prevalence of Cytomegalovirus (CMV) infection among pediatric patients with BA. The prevalence of active CMV infection within this demographic is reported to range from approximately 30% to as high as 64%, underscoring the potential significance of CMV in the pathogenesis of BA (Shen et al., 2008; Fischler et al., 2022). Significant evidence suggests a potential role for rotavirus in the development of BA. However, definitive proof is yet to be established. Infection of Ross River virus (RRV) soon after birth is a well-established biliary epithelium injury model and shares human BA’s phenotypic features, which demonstrates perinatal viral infection as the possible cause of its pathogenesis (Averbukh and Wu, 2018). In addition to CMV and rotavirus, rhinovirus, human herpesvirus, human oncovirus, adenovirus, Epstein-Barr virus, hepatitis B virus, and parvovirus B-19 are also involved in the pathologic process of BA (Saito et al., 2015). While the specific mechanisms of viral-induced BA remain elusive, one central pathophysiological concept involves immune dysregulation triggered by viral infection. Viruses may provoke an immune response by directly harming bile duct epithelial cells or indirectly by stimulating bile ducts and the liver, leading to the release of inflammatory mediators such as pro-inflammatory cytokines (TNF-α, IL-6, IL-1β) and chemokines(CX3CR1), which are thought to contribute to the development of BA (Bezerra et al., 2018; Wang et al., 2020b).

### Immune dysregulation

The pathogenesis of BA implicates a complex interplay between immune dysfunction, autoimmunity, and the inherent susceptibility of the neonatal immune system, which is notably immature (Klemann et al., 2016; Wang et al., 2020b). While research has begun to elucidate the dynamic interplay between innate and adaptive immune responses in the development of BA, the precise regulatory mechanisms still need to be fully understood (Wang et al., 2020b). BA, a severe pediatric liver disease, has been linked to perinatal viral infections that can disrupt immune regulation (Saito et al., 2015). Following such infections, a cascade of chemokines, including CXCL2, is released (Jee et al., 2022; Wang et al., 2023). These chemokines facilitate the differentiation of T helper 1 (Th1) cells and stimulate the secretion of interleukin-2 (IL-2) and interferon-gamma (IFN-γ) (Brindley et al., 2012). The subsequent activation of cytotoxic T lymphocytes (CTLs) can directly destroy the bile duct epithelium (Brindley et al., 2012). Additionally, these events can activate Kupffer cells, resulting in a significant infiltration of inflammatory factors within the liver (Wang et al., 2020b). This inflammatory response can cause damage to both the biliary epithelium and hepatocytes (Wang et al., 2023). Concurrently, the activation of hepatic stellate cells (HSCs) is triggered, leading to the secretion of collagen and the promotion of liver fibrosis (Davenport et al., 2001). Moreover, viral infections can activate the complement system, mediating direct damage to the biliary epithelium by natural killer (NK) cells (Miethke et al., 2010; Wang et al., 2020b). Recent studies have identified a role for B-lymphocytes in the pathogenesis of BA, with histopathological examinations in pediatric patients revealing an accumulation of IgG-associated antibodies within the liver (Wang et al., 2020b). This finding suggests a contribution of autoimmunity to the disease process. Regulatory T (Treg) cells are increasingly recognized as pivotal in the pathogenesis of BA (Brindley et al., 2012). In a murine model constructed using the RRV, a depletion of Treg cells in the liver was observed. However, the prophylactic intraperitoneal injection of Treg cells from wild-type mice into neonates prior to RRV infection significantly improved both the pathological phenotype and the survival rate of the mice. Subsequent research has elucidated that Treg cells ameliorate BA by curbing the overactivation of CD8^+^ T lymphocytes (Muraji et al., 2008; Miethke et al., 2010). Furthermore, Treg cells have been shown to mitigate BA symptoms by reducing the number of NK cells and through the secretion of IL-17 and TNF-α (Zhang and Kang, 2022). Concurrently, γδT cells have been implicated in this immunomodulatory process (Klemann et al., 2016). These findings suggest that the immune profile of children with BA may include the innate immunity, adaptive immunity, and graft-versus-host immunity, among others. Understanding these immune features may reveal potential targets for novel therapeutic interventions for BA.

### Genes and heredity

Numerous clinical studies have revealed that racial disparities have long been recognized in BA and are explained by intrinsic genetic predisposition and other environmental factors (Hartley et al., 2009). The incidence of BA is less prevalent in Europe and The United States, but it is relatively more common in Asia (Sanchez-Valle et al., 2017; Schreiber et al., 2022). Multiple genome-wide association studies have identified a risk locus on chromosome 10q24.2 in Asian and white cohorts (Garcia-Barceló et al., 2010). The adducin three gene (ADD3), which is located in this region, has been investigated, and it has been found that variants that alter ADD3 expression affect the outcomes of BA. Furthermore, the deletion of ADD3 in a zebrafish model leads to abnormal biliary tract (Cui et al., 2023). These findings suggest that the occurrence of BA might be related to ethnogenetic factors. It has also been observed that BA runs in families. In clinical cohort studies of BA, a significant familial clustering of BA has been observed, with three out of five children in a given family developing BA (Tran et al., 2021). Unfortunately, the mechanisms by which genetic factors contribute to the development of BA are still unknown. Genetic mutations also play a crucial role in the development of BA. Defects in specific genes, such as AMER1, INVS, and OCRL, have been detected in children with BA combined with malformations (Tran et al., 2021). Additionally, a recessive mutation in the inv gene was found to cause persistent jaundice followed by atresia of the extrahepatic bile ducts in mice, which is highly similar to the pathologic changes of BA in human beings (Mazziotti et al., 1999). This finding suggests that genes play an essential role in the development of BA.

### Drug and toxin

Drugs and toxins are also involved in the occurrence of BA. In several outbreaks of BA in lambs and calves in Australia, it was found that pregnant lambs and calves were exposed to a new type of isoflavone toxin (biliatresone) (Harper et al., 1990; Lorent et al., 2015). Subsequently, in animal models of mice, it was confirmed that biliatresone could cause serious destruction of the extrahepatic biliary tree and lead to the loss of cilia in the bile duct cells of newborn mice (Yang et al., 2020). This suggests that environmental toxins may be associated with certain BA cases. Hosoda et al (Hosoda et al., 1997). gave pregnant Wistar rats intraperitoneal phalloidin (an actin-binding toxin). Hepatic histology showed fibrosis, thickening of the extrahepatic bile duct wall, and stenosis and atresia of the duct lumen. The liver showed interlobular fibrosis, and, like human BA, complete occlusion was found only in rats exposed to the drug during the fetus. In mouse cholangiocytes, the toxin biletriaxone was found to reduce glutathione levels by inhibiting GSH synthesis. This reduction in GSH levels and the presence of thione and SOX17 led to extrahepatic cholangiocyte injury and fibrosis in mice, as observed in three-dimensional spheroid cultures and neonatal extrahepatic duct explants (Waisbourd-Zinman et al., 2016). In summary, drugs and toxins play an important role in the development of the BA.

### Gut microorganisms and their metabolites

An increasing body of evidence has shown that gut microbiome can impact the occurrence of BA because of its induction of persistent intestinal inflammation and intestinal barrier function (Jee et al., 2017; Wu et al., 2024). A clinical study demonstrated marked differences in the gut microbiome structure between patients with BA and healthy controls, especially regarding microbial abundance and diversity (Wang et al., 2020a; van Wessel et al., 2021; Chen, 2022; Sun et al., 2022). BA showed lower diversity and significant structural segregation in the microbiome. At the phylum level, *Proteobacteria* numbers increased, whereas those of *Bacteroidetes* decreased in BA. At the genus level, several potential pathogens, such as *Streptococcus* and *Klebsiella*, thrived in BA, while numbers for *Bifidobacterium* and several butyrate-producing bacteria declined (Han, 2019; Chen, 2022). Increases in harmful bacteria elevate the probability of direct interaction between the microbes and host tissue. Moreover, recent reports have shown that BA is associated with gut microbiota dysbiosis, disrupted intestinal barrier, and chronic inflammation (Alexander et al., 2023; Wu et al., 2024). In several experimental studies related to BA, it has been found that the intestinal barrier is differentially damaged in children with BA (Abu Faddan et al., 2017; Yan et al., 2022). This is evidenced by increased lipopolysaccharide (LPS) levels in the whole body and liver and significant hepatic inflammatory responses triggered by LPS (Isayama et al., 2006; Yan et al., 2022). In the RRV mouse model, signs of intestinal barrier damage were observed, including decreased mucosal thickness, glandular crypt depth, and villus height. In the rat model of BA with choledochal ligation, the intestine showed edema and inflammatory lesions, formation of intestinal epithelial gaps, reduction of tight junctions, decreased secretory immune proteins, and increased bacterial adherence to the mucosa. These data suggest that BA destroyed the gut microbiome’s structure, damaged the intestinal barrier’s function, and increased intestinal permeability (Jee et al., 2017). Cholangitis following KPE also offers compelling evidence for the translocation of gut bacteria (Wang et al., 2023). The isolation of systemic bacteria with intestinal origins, such as *Klebsiella* and *Escherichia coli*, in pediatric cholangitis cases underscores the link between the gut microbiota and the pathogenesis of BA (Song et al., 2021b; Zheng et al., 2022). This suggests that the gut microbiota may play a pivotal role in the development of BA, potentially through the disruption of the gut barrier function, leading to bacterial and PAMP translocation.

Not only is the gut microbiota closely related to the host’s health, but the metabolites of the gut microbiota also play an important role. short-chain fatty acids (SCFAs), particularly butyrate, have shown positive effects such as promoting anti-inflammation and enhancing intestinal epithelial barrier function through binding and activating SCFAs receptors (Sokal et al., 1996). Xu et al (Xu et al., 2023). found that changes in the gut microbiota composition in BA, especially the butyrate-producing microbiota, and butyrate levels in BA were negatively correlated with jaundice clearance and cholangitis (Sokal et al., 1996). A recent large‐scale metabolomics analysis showed that BA is associated with a signature in amino acid metabolites (Zhou et al., 2015). Unfortunately, the current study did not examine the crosstalk between metabolites and gut microbiota for the BA. These findings suggest that the composition of the gut microbiota and their associated metabolites play an essential role in the pathogenesis of BA, and gut microbiota alterations may serve as potential therapeutic targets in the future.

### Other factors

Other factors could also play a role in the procession of these particular BA. Vascular abnormalities may be one of the causes of BA. An analysis conducted by Ho et al (Ho et al., 1993). on 11 cases of BA revealed hyperplasia and hypertrophic tortuous hepatic artery branches in all patients’ extrahepatic and intrahepatic parts. Histological examination of the liver of BA children suggested ischemic and hypoxic factors in the portal area of the liver. Vascular endothelial growth factor was found to promote BA and positively correlated with the degree of liver fibrosis. This indicates that vascular abnormalities may be involved in the development of BA. Additionally, some scholars have suggested that maternal microchimerism may be a pathogenesis of BA (Muraji et al., 2008). Some studies have shown that maternal cells containing X chromosomes have been found in the liver of male BA children. These X chromosome-containing cells also express CD8^+^ or cytokeratin, suggesting that maternal cells may be immune cells that can participate in developing biliary epithelium. Thus, maternal microchimerism may be a potential pathogenesis of BA (Muraji et al., 2008).

## Alterations in the gut microbiota and metabolites associated with biliary atresia

BA is an occlusive fibroinflammatory biliary disease affecting the bile ducts in infants. It is characterized by rapid liver fibrosis progression, with histopathological changes including ductal reaction, portal fibrosis, and bile thrombi (Bezerra et al., 2018; Shen et al., 2019). Ductal reaction manifests as small bile duct proliferation around portal areas, while portal fibrosis is defined by the formation of fibrous septa between liver nodules. Liver fibrosis in BA involves the production of extracellular matrix (ECM) by activated hepatic stellate cells (HSCs), a wound-healing response (Shen et al., 2019). Bile acids have cytotoxic effects that activate HSCs and stimulate the secretion of profibrogenic mediators, such as transforming growth factor beta, reactive oxygen species, TNF-α, and platelet-derived growth factor (Waisbourd-Zinman et al., 2016). These factors enhance HSC activation and ECM synthesis, exacerbating fibrosis beyond that seen in other pediatric and adult diseases (Kisseleva and Brenner, 2021). Timely intervention is crucial as untreated BA can progress to cirrhosis within weeks of birth (Bezerra et al., 2018). However, due to persistent jaundice and recurrent cholangitis, liver fibrosis continues to progress swiftly, resulting in a long-term survival rate of approximately 50% for children with BA (Schreiber et al., 2022). Post-surgery progressive liver fibrosis has become the primary constraint on the efficacy of KPE. Consequently, many patients eventually require an LT as a life-saving procedure (Hartley et al., 2009; Sanchez-Valle et al., 2017; Bezerra et al., 2018). The international shortfall between available donor organs and the number of patients requiring a transplant is significant, meaning patients often deteriorate and succumb while awaiting a transplantation (Zarrinpar and Busuttil, 2013).

**Figure 2:**
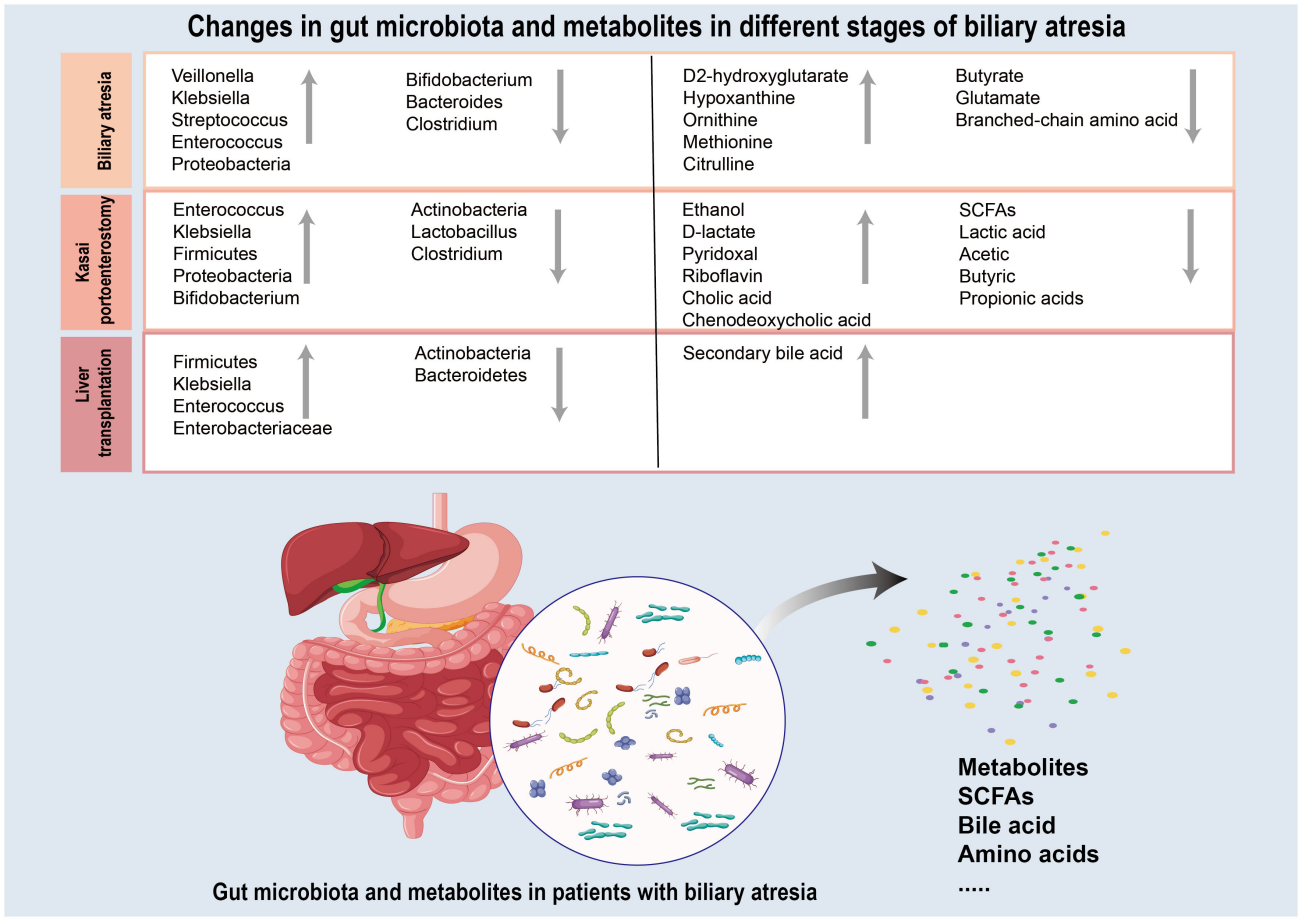
Summary of the gut microbiota and metabolites changes during the three different stages (biliary atresia period, Kasai hepatoportoenterostomy period and liver transplantation period) of biliary atresia. Grey upward arrows represent gut microbiota and metabolites whose level is increased in biliary atresia relative to those in healthy control. Grey downward arrows represent gut microbiota and metabolites that are decreased in biliary atresia relative to those in healthy control.

Gut microbiota and their metabolites has been suggested to play a role in almost all major diseases including hepatobiliary diseases. The gut microbiota interacts with the liver and gallbladder through different mechanisms, such as diet, bile acids, immune factors, and metabolites, including SCFAs, to maintain intestinal homeostasis (Jain et al., 2021). The gut microflora has been implicated in the development and progression of BA. Particular microbiome diversity is highly variable in patients with BA, and the characteristics of the gut microbiota vary at different stages of BA(**Figure 2**) (Han, 2019; Qin et al., 2021; Chen, 2022; Fu et al., 2022a). Gut microbiota and their metabolites may participate in regulating immune response during inflammation, tissue repair following damage and autoimmunity and release a variety of pro-inflammatory and chemotactic factors to promote hepatic fibrosis through the degradation of stromal collagen and regulation of HSCs, thus improving the course of BA (Davenport et al., 2001; Wang et al., 2020b). Understanding the gut microbiota and metabolic changes in different BA stages could lead to novel therapeutic approaches for this condition (**Tables 1**, **2**).

**Table 1:** Dysbiosis of gut microbes and metabolite in biliary atresia.

Patient/Model	Dysbiosis of the Microbes	Metabolite Changes	Ref
Biliary atresia without treatment			
43 BA patients and 22 HC	*Streptococcus, Klebsiella* and *Haemophilus* ↑↑; *Bifidobacterium, Bacteroides* and *Lactobacillus* ↓↓	NA	(Chen, 2022)
11 BA patients and 10 HC	*Veillonella* ↑↑; *Bacteroides* and *Clostridium* ↓↓	NA	(Han, 2019)
55 BA infants, 19 HC and 21 cholestatic control	*Enterococcus* and *Clostridium* ↑↑; *Bifidobacterium*↓	NA	(Jain et al., 2023)
102 BA infants and HC	*Proteobacteria*, *Bacilli* (*Lactobacillus*), *Fusobacteria*, and *Streptococcus*, *Klebsiella*, and *Enterococcus* ↑↑ *Bacteroides* and *Clostridium* ↓↓	Hypoxanthine ↑; glutamate/glutamine↓	(Jee et al., 2022)
9 bottle-fed infants with BA and HC	*Bifidobacteria*, lecithinase-negative *clostridia*, *Streptococci*, and *Staphylococci*↓↓	NA	(Kobayashi et al., 1988)
35 BA patients, 35 patients with neonatal cholestasis rather than BA, and 35 HC.	NA	Ornithine, methionine, and citrulline ↑; Glycine, branched-chain amino acid ↓	(Raouf et al., 2016)
16 BA (early stage) patients, 16 HC;16 BA (later stage) patients, 10 HC	*Proteobacteria*, *Klebsiella*, *Streptococcus*, *Veillonella*, and *Enterococcus*↑↑ *Bifidobacterium*, *Actinobacteria*, *Verrucomicrobia* and *Blautia*↓↓	NA	(Song et al., 2021b)
46 BA patients and 22 HC	NA	D2-hydroxyglutarate	(Tian et al., 2022)
34 BA patients and 34 HC	*Streptococcus* and *Klebsiella*↑↑ *Bifidobacterium*↓↓	Butyrate ↓	(Wang et al., 2020a)
43 BA patients, 33 other cholestatic diseases and 42 HC	*Proteobacteria*, *Streptococcus* and *Lactobacillus*↑↑	Butyrate ↓	(Xu et al., 2023)
Biliary atresia with KPE			
30 BA children and 23 HC	*Enterococcus*↑↑	SCFAs, lactic acid, acetic, butyric, and propionic acids↓↓	(Orłowska et al., 2021)
12 pre-LT, 12 BA post-LT and 22 HC	*Firmicutes* ↑↑ *Actinobacteria*↓↓	NA	(Chen, 2022)
40 BA patients	*Bifidobacterium* and *Lactobacillus* ↑↑ *Faecalibacterium*↓↓	NA	(Fu et al., 2022a)
40 BA patients	*Escherichia coli.* ↑↑ *Bifidobacterium* and *Lactobacillus*↓↓	NA	(Fu et al., 2022b)
55 BA infants pre-KPE	NA	Ethanol and D-lactate ↑↑	(Alexander et al., 2023)
4 BA patients	*Proteobacteria*↑↑	NA	(Elaine Chen et al., 2020)
55 BA infants, 19 HC and 21 cholestatic control	*Enterococcus*↑↑ *Bifidobacterium*↓↓	Acetate↓↓	(Jain et al., 2023)
39 BA patients	*Klebsiella*↑↑	NA	(Meng et al., 2021)
16 BA patients (8 non-KPE, 8 post-KPE)	*Bacteroides*, *Prevotella*, *Barnesiella*, *Parabacteroides*, *Heliobacterium*, *Erysipelatoclostridium*, and *Diaporthe*	Pyridoxal and riboflavin ↑↑ cholic acid, chenodeoxycholic acid, and β-muricholic acid↑↑	(Song et al., 2021a)
8 BA patients and 7 cholestasis	*Bifidobacterium* and *Enterobacter*↑↑ *Enterococcus*↓↓	NA	(Tessier et al., 2020)
Biliary atresia with LT			
12 pre-LT, 12 BA post-LT and 22 HC	*Firmicutes*↑↑ *Actinobacteria* and *Bacteroidetes*↓↓	NA	(Chen, 2022)
16 BA patients and 10 HC	*Klebsiella*, *Enterococcus*, *Enterobacteriaceae Bacterium*↑↑	NA	(Song et al., 2021c)
10 BA patients	*Klebsiella*↑↑	Secondary bile acid↑↑	(Waldner et al., 2023)

'↑' and '↓' indicate increases and decreases, respectively, compared to the reference or baseline value. 'NA' stands for 'Not Applicable', indicating missing or not relevant data for the specific item.

**Table 2:** Metabolites and their impact on functions in biliary atresia.

Metabolite	Source Bacteria	Impact on Functions	Proposed Role in BA	Ref
Butyrate	*Proteobacteria*, *Streptococcus* and *Lactobacillus*	Jaundice clearance and cholangitis	Noninvasive diagnostic i indicator	(Xu et al., 2023)
Secondary bile acid	*Klebsiella*	Cholangitis and Complications after LT	Guidance on the use of immunosuppressive agents	(Waldner et al., 2023)
Acetate	*Bifidobacterium*	Cholangitis	Possible protective factor	(Jain et al., 2023)
Propionate	*Clostridium*	Total bilirubin	Possible protective factor	(Jain et al., 2023)
glutamate/glutamine and hypoxanthine	*Bacteroidetes* and *Clostridia*	Promoting survival of bile duct epithelial cells	Potential Treatment Options	(Jee et al., 2022)
Tauroursodeoxycholic acid	*Campylobacter* and *Rikenellaceae*	Jaundice clearance	Potential Treatment Options	(Yang et al., 2022)
Bile acids	*Faecalibacterium prausnitzii* and *Escherichia coli*	Maintaining the intestinal barrier	Possible protective factor	(Song et al., 2021a)
Tryptophan	*V. atypica*	Total bilirubin	Noninvasive diagnostic i indicator	(Song et al., 2021b)
Lithocholic acid	*Enterococcus faecium*	Total bilirubin	Noninvasive diagnostic i indicator	(Song et al., 2021b)
Branched chain amino acids	NA	Correct growth, nitrogen retention, and body composition	Potential Treatment Options	(Sokal et al., 2021b)

### Gut microbiota and metabolic in patients with biliary atresia

Studies have confirmed altered gut microbiota richness and diversity indices in BA patients (Han, 2019; Wang et al., 2020a). There were significant differences in the composition of intestinal microorganisms between the BA group and the control group. From the phylum level perspective, the relative abundance of Bacteroidetes in children with BA was significantly lower than that in the control group. In contrast, the abundance of Firmicutes was increased considerably. At the genus level, the relative abundance of Bacteroides and Clostridium in the feces of children with BA was significantly reduced. In contrast, the relative abundance of Veillonella was considerably higher than that of the control group (Wang et al., 2020a; Chen, 2022). One of the typical characteristics of BA is a decrease in fecal bile acids, which are synthesized in the liver from cholesterol, converted into primary bile acids, and metabolized in the intestine via the enterohepatic circulation (Yan et al., 2022; Yang et al., 2022; He et al., 2023). Yang et al (Yang et al., 2022) found that Bifidobacterium showed a significant negative correlation with conjugated bile acids (GCA, TCA, TCDCA, and GCDCA; all p < 0.05), and Rothia was negatively correlated with secondary bile acids (DCA and LCA; both p < 0.05). Unfortunately, current research cannot confirm the relationship between bile acids and gut microbiota. The interaction between the gut microbiota and bile acids is complex and bidirectional in the host and is referred to as the gut microbiota-bile acid axis (Cai et al., 2022). The gut microbiota converts primary bile acids into secondary bile acids, suggesting that the gut microbiota can affect the composition of secondary bile acids. Changing the secondary bile acid profile could reshape the intestinal bacterial composition and maintain gut homeostasis (Tilg et al., 2022; Zhang et al., 2022; He et al., 2023). The current view is that BA may result from a combination of factors, including genetics, the environment, infections during pregnancy, and immunologic factors (Ho et al., 1993; Bezerra et al., 2018). Dysbiosis of the gut microbiota and abnormal bile acid metabolism may be two of these complex factors, which may play a superimposed contributory role in the development of BA but are unlikely to be the sole cause. Therefore, it is not accurate enough to consider dysbiosis of the gut microbiota or abnormal bile acid metabolism alone as a single cause of BA. Future studies need to elucidate further how these factors interact and their specific roles in the pathogenesis of BA.

Not only is the gut microbiota closely related to the health of the host, but the metabolites of the gut microbiota also play an essential role in the process of BA (Cai et al., 2022). Recently, owing to the development of transcriptomics and metabolomics technology, more research has reported changes in the production of bacterial metabolites, including SCFAs, bile acids, and amino acids in BA patients (Song et al., 2021a; Yang et al., 2022). SCFAs, such as butyric, propionic, acetic, and valeric acids, are carboxylic acids produced through aerobic fermentation of dietary fiber in the intestine (Rauf et al., 2022). It is well known that SCFAs have an essential function in maintaining human health, including gut homeostasis, and serving as the primary fuel for the colonic epithelial cells (Qin et al., 2021). SCFAs display anti-inflammatory activity and are responsible for strengthening the intestinal barrier, which prevents gut leaks (Yan et al., 2022). Due to these properties, they are particularly beneficial in patients with intestinal inflammation. Among SCFAs, butyric acid has the best proven beneficial effect, whereas the importance of the other acids still needs to be understood. In addition to SCFAs, the leading bacterial products, the gut microbiota forms other organic acids. Their role has yet to be discovered. Among them, we distinguish isobutyric and isovaleric acids, collectively called branched short-chain fatty acids(BCFAs), produced through the fermentation of branched-chain amino acids (Rauf et al., 2022). In contrast to SCFAs, increased BCFAs unfavorable affect gut health (Sokal et al., 1996). However, the relationship between the role of individual bacterial-derived acids and intestinal inflammation remains unclear. Moreover, the association between factors influencing BA onset and course, such as eating habits, lifestyle, medications, and the profile of bacterial-derived acids, still needs to be fully understood.

### Gut microbiota and metabolites of biliary atresia patients treated with Kasai surgery

Since 1959, KPE has been recognized as a first-line treatment option for BA. This procedure involves removing a portion of the fibrous mass caused by hepatic portal BA and establishing a hepatico-jejunal anastomosis to restore bile drainage (Sanchez-Valle et al., 2017). KPE has demonstrated prompt relief of bile flow obstruction and improved chances of long-term survival without the need for LT (Bezerra et al., 2018). Clinical studies have shown that an imbalance of gut microbiota exists in children before KPE, with a decrease in the abundance of *Bifidobacteria* and LGG and an increase in the abundance of aerobic bacteria such as *Escherichia coli* and *Enterococci* spp. which is even more pronounced in the early postoperative period (Han, 2019; Song et al., 2021b; Chen, 2022; Jain et al., 2023). Fu et al (Fu et al., 2022b). studied the effect of early nutritional support on the intestinal flora of children after KPE. They found that 7 d after KPE, the levels of *Bifidobacteria* and LGG decreased significantly, and the levels of *E. coli* and *Enterococci* spp. increased significantly compared with those before KPE. In 2022, Fu et al (Fu et al., 2022a). conducted another study on the intestinal flora of children after KPE. They found that the levels of *Bifidobacteria* in children with BA were higher than that of pre-KPE, the levels of *E. faecalis* were lower than that of pre-KPE, and the levels of E. faecalis were lower than that of pre-KPE in children with BA. The LGG level was lower than pre-KPE, and the level of LGG gradually increased in the late postoperative period after KPE. This shows a significant difference in the composition of the gut microbiota at different stages after KPE. This difference may be due to surgical stress, postoperative application of antibiotics and hormones, and changes in bile acid metabolism in the intestinal tract. Anesthesia and surgery (especially gastrointestinal surgery) are potent stimulants for the body, which can induce a severe peripheral inflammatory response that can affect various organs and systems throughout the body, including intestinal tissues. Anesthetic drugs and surgical trauma can affect the gut microbiota (Çitoğlu et al., 2012; Micó-Carnero et al., 2020). In addition, antibiotics play an essential role in preventing postoperative infections. However, it is also an important factor in the dysbiosis of the gut microbiota after KPE.

While KPE can effectively alleviate symptoms of cholestasis and improve short-term prognosis in certain children with BA, it is important to note that progressive hepatic fibrosis and recurrent cholangitis are common complications that may arise post-surgery (Bezerra et al., 2018). Cholangitis, in particular, is a frequent occurrence post-KPE, and its underlying cause in BA is still unclear. However, retrograde infection by intestinal bacteria, including *Pseudomonas aeruginosa*, *Escherichia coli*, *Enterobacter cloacae*, *Klebsiella*, *Acinetobacter baumannii*, and *Salmonella typhimurium*, are believed to be the primary cause (Madadi-Sanjani et al., 2021; van Wessel et al., 2021).

Metabolites of the gut microbiota have been shown to play an vital role in the progress of BA disease. Recent studies have shown significant metabolic abnormalities in post-KPE in BA (Zhou et al., 2015; Jain et al., 2023). Orowska et al (Orłowska et al., 2021). analyzed the postoperative fecal composition of patients undergoing KPE surgery. They found significant differences in the expression of several metabolites in patients treated with LGG compared to those in the placebo group. Moreover, patients with postoperative cognitive impairment showed decreased levels of SCFAs, lactic acid, acetic, butyric and propionic acids. Previous studies have confirmed that increased levels of SCFAs alleviate the extent of liver injury (Rauf et al., 2022). Alexander et al (Alexander et al., 2023). found that Ethanol, at three months post-KPE, is associated with LT in BA. Ethanol and D-lactate production are linked, suggesting a role for gut microbiota-ethanol and D-lactate production in BA. Jain et al (Jain et al., 2023). observed 55 cases of BA. They found that a decrease in *blautia*, *bifidobacteria*, and subsequent dysregulation of SCFAs in the early post-KPE period related to poorer clinical outcomes. In addition, increased intestinal permeability and decreased Acetate levels were found in the early post-KPE. Although several studies have described the current status of alterations in the gut microbiota and its metabolites in children with BA treated with KPE, the specific mechanisms involved in their occurrence remain to be investigated, and thus, more basic and clinical studies are still needed in the future to more systematically investigate the potential mechanisms of action of the gut microbiota and its metabolites in children with BA treated with KPE.

### Gut microbiota and metabolites in biliary atresia patients undergoing liver transplantation

KPE effectively alleviates the symptoms of cholestasis and improves the quality of life of patients with BA. Despite these benefits, BA remains the primary reason for liver transplantation (LT) in the pediatric population due to the persistence of progressive cholestasis and recurrent cholangitis even after KPE treatment (Hartley et al., 2009; Bezerra et al., 2018). Following LT in children with BA, various factors such as ischemia/reperfusion injury, postoperative infections, and chronic rejection can have an impact on the microbiota composition (Micó-Carnero et al., 2020). The administration of immunosuppressants, antibiotics, antifungal and antiviral drugs post-transplantation can further disturb the balance of gut microbiota, gastrointestinal epithelial barrier function and increased gut permeability, and result in bacterial translocation (Micó-Carnero et al., 2020; Qin et al., 2021; Sucu et al., 2023). Furthermore, Studies have confirmed that gut microbiota and their metabolites correlate with prognosis and complications after LT (Madadi-Sanjani et al., 2021; Wirth et al., 2023). These findings underscore the critical role of microbiota composition in LT outcomes.

The diversity and number of microorganisms in the gut microbiota of children with BA undergoing LT are significantly altered, with a decrease in the number of beneficial bacteria and an increase in the number of conditional pathogens in the early stages, and a gradual recovery of the microbiome diversity in the late stages, with a decrease in the *Enterobacteriaceae* and a gradual increase in the number of potentially beneficial bacteria, such as the *Rumatobacteriaceae (* Yao et al., 2023). Chen et al (Chen, 2022). conducted a prospective study analyzing the microbiome diversity of 12 patients underwent LT in preoperative and early postoperative periods. There is a significant shift in the diversity of gut microbiota during the early post-LT period, with an increase in the abundance of the *Firmicutes* and a decrease in the abundance of the *Actinobacterial* and *Anaplastic bacterial phyla* compared to the preoperative period. In an animal model of LT, the researchers found that rats had a significant decrease in the abundance of probiotics, such as *Bifidobacterium* and LGG, and a significant increase in the abundance of conditional pathogens, such as *Enterobacteriaceae* and *Enterococcus* spp., after undergoing LT. This change in microbiota composition was associated with functional changes related to increased pathogenic toxins in the early postoperative period and perioperative stress. Song et al (Song et al., 2021b). evaluated fecal samples from 16 children with BA who underwent LT pre-transplantation, as well as six months post-transplantation and the fecal microbiomes of 10 healthy controls and found that the microbiome diversity of the children with BA increased at six months post-LT. The differences in microbiota composition were not statistically significant in the post-LT group when compared to healthy controls, suggesting that LT restores, at least partially, the microbiota composition. Similar results were obtained by Waldner et al (Waldner et al., 2023). analyzed the microbiome diversity in patients before and 3, 12, and 24 months after LT and found that at three months after LT, the intestinal alpha diversity of the gut microbiota was significantly reduced at three months after LT. In contrast, no significant difference was observed at 12 and 24 months compared to healthy controls. In addition, changes in the microbiome diversity were associated with alterations in bile acid synthesis secondary to LT and the choice of different immunosuppressive agents two years after LT. These results suggest that the diversity of gut microbiota in children with BA after LT is associated with the prognosis of LT. Current clinical and basic trials of the gut microbiota and its metabolites in children with BA treated with LT are ongoing, and it needs to be clarified how the metabolites of the gut microbiota of children with BA before and after LT are characterized.

## Roles of the gut microbiota dysbiosis on biliary atresia occurrence and development

**Figure 3:**
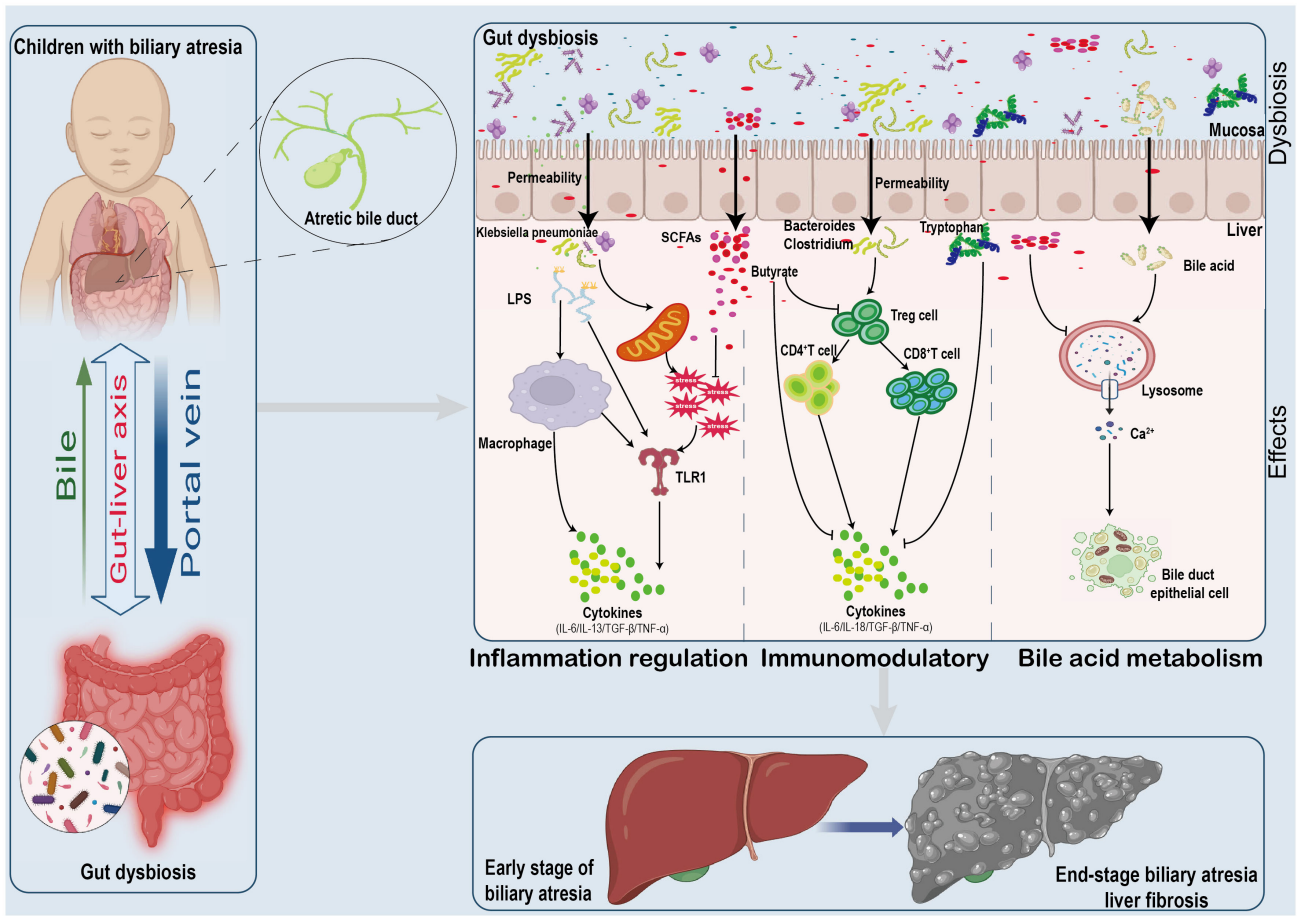
Mechanisms Linking Gut Microbiome Alterations to Biliary Atresia Progression. The gut microbiome's impact on biliary atresia involves:(1) Increased pathogen presence and metabolite release disrupting gut integrity. (2) Metabolic and cytokine shifts affecting oxidative stress and inflammation. (3) Reduced butyrate levels leading to immune imbalances and cytokine surges. (4) Bile acid metabolism alterations contributing to cellular damage and apoptosis.

The exact cause of BA is unknown; it is a multifactorial disease, such genetic, environmental factors, and intestinal immune dysfunction (Hartley et al., 2009). Recent research has shown that BA is caused by changes in the biliary, hepatic, and intestinal tracts, leading to an immune-mediated inflammatory response that causes liver injury and liver fibrosis (Han, 2019). The causes of liver immune imbalance and excessive inflammatory response can be endogenous or exogenous, ranging from hereditary factors to viral infections, environmental toxins, and adverse drug reactions. Recent clinical studies indicate that gut microbiota and their metabolites, such as SCFAs (acetate and butyrate) and ethanol, are crucial in the pathogenesis of BA (**Figure 3**) (Jee et al., 2017).

### Gut microbiota and metabolites participate in the development of biliary atresia by regulating inflammation

Inflammatory response plays an essential role in the development of BA. Pathological examination of the liver in children with BA reveals that the hepatic portal vein area is infiltrated with many inflammatory cells, including NK cells, CD4^+^ T cells, CD8^+^ T cells, B cells, and macrophages (Han, 2019; Chen, 2022). Activated inflammatory cells secrete many inflammatory mediators, such as interferon-gamma, tumor necrosis factor-α(TNF-α), and interleukins(IL). These cytokines further damage the bile duct epithelial cells, causing obstruction, liver fibrosis, and cirrhosis (Brindley et al., 2012). Studies have confirmed that gut microbiota can influence the condition of children with BA by participating in the body’s inflammatory response (Çitoğlu et al., 2012). Meng et al (Meng et al., 2021). found that the postoperative KPE induced gut microbiota dysbiosis, as reflected by reduced diversity. Moreover, the relative abundance of *Klebsiella* increased in the fecal. Gene expression patterns in BA-like organs transfected with *Klebsiella*. Infections were enriched in pathways associated with inflammation, apoptosis and fibrosis. Further studies revealed IL-13/TGF-β1 mediated fibrosis in postoperative cholangitis in KPE. This study suggests the involvement of *Klebsiella* in regulating the inflammatory response, which further influences the process of BA.

Metabolites of gut microbiota are directly involved in energy metabolism locally but also reach the liver via the liver-gut axis to modulate the inflammatory response (Milosevic et al., 2019; Tilg et al., 2022). Therefore, disturbed gut microbiota can alter hepatic inflammation by affecting metabolite production, among which SCFAs, metabolites of gut microbiota, have been widely recognized. SCFAs were found to exert anti-inflammatory properties by activating G protein-coupled receptors GPR41 and GPR43 and also regulate the production of pro-inflammatory factors, such as IL-6 and TNF-α, through the TLR1 pathway in the Toll-like receptor, to alleviate the systemic inflammatory response (Rauf et al., 2022). *Eicosapentaenoic acid* inhibited liver fibrosis in patients without jaundice six months after KPE. Further studies found that periductal inflammation was reduced by *Eicosapentaenoic acid* supplementation (Zhou et al., 2015). Thus, metabolites of the gut microbiota can be involved in inflammatory regulation, thereby influencing the pathological process of BA. In addition, structural dysregulation of the gut microbiota is also involved in the regulation of inflammation in the bile ducts in the Cxcr2^-/-^ mouse model of RRV infection model showed further enrichment of *Corynebacterium*, *Anaerococcus* and *Streptococcus (* Jee et al., 2017). Among these, *Anaerococcus lactolyticus* was significantly associated with a suppression of biliary injury, cholestasis, and survivability, leading to an inflammatory response and oxidative stress, which are involved in the pathological process of BA.

### Gut microbiota and metabolites participate in the progression of biliary atresia by regulating immune response

The modulation of abnormal immune regulation is one of the possible mechanisms in the pathogenesis of BA. Many immune cells are involved in the development of BA. Brindley et al (Brindley et al., 2012). found that the number of Treg cells in children with BA decreased and had a significant negative correlation with the degree of liver fibrosis. Fu et al (Fu et al., 2022a). studied liver biopsies from patients with BA and found that CD4^+^ and CD8^+^ infiltrates were predominantly present in perihepatic bile duct lymphocytes. In addition, the abundance of Bifidobacterium bifidum increased significantly, and the abundance of E. faecalis decreased pre-KPE. In comparison, the level of CD4^+^ and CD8^+^ was higher than pre-KPE, and the level of IL-18 was lower. Jee et al (Jee et al., 2022). found that hepatic immune cell activation and survival traits were associated with fecal characteristics of *Mycobacterium anomalies* and *Clostridium perfringens* in the RRV-induced BA mouse model. Together, these findings illustrate the essential role of immune response in the BA course. However, the studies mentioned above did not conduct in-depth research on the regulatory mechanism, and the specific mechanism still needs to be clarified. The findings of Jee et al (Jee et al., 2017). also illustrate that butyrate can reduce liver and bile duct inflammation by defending against oxidative stresses in humans and the BA mouse model. Butyrate is a microbial metabolite that has been shown to affect the liver-gut axis, including directly scavenging free radicals and indirect antioxidant activity through modulation of the pathways involved in expressing cytoprotective enzymes and molecules. In addition to butyrate, tryptophan, which is associated with *Klebsiella*, *Veillonella*, and *Enterococcus* spp., has been shown to exacerbate liver injury in BA, either through direct or indirect effects.

### Gut microbiota and metabolites participate in the progression of biliary atresia by regulating bile acid metabolism

Bile acid metabolism disorders in BA is one of the most important causes of liver injury (Liu et al., 2015). Due to bile duct obstruction, children with BA have higher total bile acid levels and significantly elevated hydrophobic bile acid salt concentrations, which can lead to lysis of the endoplasmic reticulum of hepatocytes and bile duct epithelial cells and uncontrolled release of intracellular Ca^2 +^ into the cytoplasm, thereby inducing cell damage (Wang et al., 2022). Yang et al (Yang et al., 2022). analyzed the faeces of 84 children with BA. They found that patients with BA had different gut microbiota and bile acid composition characteristics, and their interactions were involved in the process of liver injury in BA, which was closely related to the development of postoperative cholangitis and clearance of jaundice. Wang et al (Wang et al., 2020a). also found that the presence of bile acids was dramatically decreased in BA, and Clostridium XI/Va positively correlated with the ratio of primary/secondary bile acids. Unfortunately, previous studies have not conducted an in-depth exploration of the issue. With more awareness of the relationship between gut microbiota and BA, we expect that more studies will be conducted to reveal the exact mechanism and provide new insights into the treatment of BA from the point of view of gut microbiota regulating bile acid metabolism.

## Therapeutic strategies targeting gut microbiota in biliary atresia

The current management of BA in children involves a combination of surgical and pharmacological interventions. Surgical treatment, known as KPE, aims to restore bile flow, while pharmacological therapies, including antibiotics, glucocorticosteroids, hepatoprotective agents, and nutritional support, are used with surgery (Bezerra et al., 2018; Schreiber et al., 2022). Recent clinical and experimental studies have highlighted the potential benefits of modulating the gut microbiota to enhance BA treatment outcomes (Orłowska et al., 2021; Jee et al., 2022). This includes strategies like administering probiotics, prebiotics, synbiotics, and metabolites like SCFAs. The cognition of gut microbiota and its metabolites in the progression of BA offers a new possibility to improve its therapeutic efficacy by targeting the gut microbiota.

### Probiotics and their potential benefits in biliary atresia management

The main genus of probiotics being studied is LGG, which has shown beneficial effects on BA in children when given in adequate doses. Two small clinical trials have investigated LGG supplementation. One small randomized controlled trial found no difference between LGG (n=14) and placebo (n=16) regarding jaundice, cholangitis or graft requirements over two years (Orłowska et al., 2021). A second trial randomly assigned patients to the LGG (n=10) or neomycin (n=10) groups for cholangitis prophylaxis and found no significant difference in the frequency of cholangitis between the LGG and neomycin groups (Lien et al., 2015). The above results illustrate that the use of probiotics increased the abundance of beneficial bacteria and decreased the abundance of conditional pathogens, maintaining microbiome diversity balance in BA patients while decreasing the risk of cholangitis and LT. Therefore, probiotic treatment could be a promising new therapeutic approach for treating BA patients.

### The metabolites of the gut microbiota as a novel approach in biliary atresia treatment

With the in-depth study of gut microbiota and its metabolites, more and more studies have confirmed that gut microbiota metabolites can regulate BA. The SCFAs are the major metabolites produced by gut microbiota fermenting dietary fibers, proteins, and peptides. The SCFAs, including acetate, propionate, and butyrate, constitute a significant class of bacterial metabolites derived from colonic carbohydrate fermentation (Rauf et al., 2022). Studies have shown that butyrate affects microbiome diversity and metabolites and phenotypic expression of experimental BA in neonatal mice and that glutamine promotes the survival of bile duct epithelial cells (Jee et al., 2022). In another study, eicosapentaenoic acid administration reduced Mac-2 binding protein sugar chain modified isomer and hyaluronic acid levels in the liver after KPE and improved liver fibrosis (Sumida et al., 2018). Other gut microbiota metabolic derivatives, such as D-2-hydroxyglutarate, can alleviate the processing of BA by regulating bile acid metabolism with the liver microenvironment and mammalian target of rapamycin signaling (Tian et al., 2022). Therefore, SCFAs and other gut microbiota metabolic derivatives may be considered novel and viable therapeutic agents for preventing and mitigating BA.

## Future perspective

Over the past decade, although partial studies on BA and gut microbiota and their metabolites have been conducted, we are still in the beginning stages of demonstrating the relevant role of gut microbiota in BA (Bezerra et al., 2018). So far, our theories are often based on retrospective and observational studies of small cohorts, and limited animal models restrict the research on BA and gut microbiota (Wang et al., 2020a; Schreiber et al., 2022). We do not have enough evidence to definitively prove that ecological dysbiosis causes BA or whether it only occurs during BA, which results from disease-related metabolic changes. Testing this hypothesis will require longitudinal, interventional, and prospective multicenter studies. Second, the specific mechanism by which specific bacterial strains or metabolic derivatives are associated with the onset of BA is unknown, and whether it can be used as an effective treatment has yet to be mechanistically determined. Further in-depth study of this specific molecular mechanism is needed in the future, which is vital for understanding the gut microbiota in the clinical diagnosis and treatment of BA. Thirdly, other microbiological factors still need to be better understood, such as the crosstalk between gut microbiota, which needs improvement. To date, we still need to confirm the sequential order of bile acids and gut microbiota alterations. The results of the current study are even contradictory. This may be due to the relatively small number of patients and the need for harmonized methods and criteria. Therefore, in-depth studies of these metabolites and their microbial sources are needed, as well as refinement of the characterization of the gut microbiota and their metabolites at different stages of BA. Technological and research developments may facilitate the use of gut microbiota in BA intervention or treatment, and in-depth exploration of the composition and function of gut microbiota will lead to new diagnostic tools and personalized treatments for BA.

## Conclusion

BA is a progressive fibroinflammatory disorder of infants involving the extrahepatic and intrahepatic biliary tree. Even with successful KPE, most patients diagnosed with BA progress to end‐stage liver disease, necessitating an LT for survival. Numerous studies have reported that gut microbiota are involved in the pathogenesis of many diseases, especially liver disease. Therefore, human biology should not neglect the gut microbiota, which produce or regulate various chemicals and trigger host responses that affect various functions, including inflammation, immunity, and metabolism. This review provides some evidence that further enhances our understanding of BA from the perspective of gut microbiota and their metabolites while simultaneously exploring the pathogenesis of BA. We also summarize the characteristics of changes in the microbiota and its metabolites at different stages in patients with BA, which may be related to the body’s inflammatory response, abnormalities in immune regulation, or the severity and progression. However, research on the in-depth mechanism of an intricate interplay between gut microbiota, metabolites, and BA pathophysiological progression remains limited, which makes any conclusive or pathogenic statements about the composition of the gut microbiota and metabolite profiles of BA patients challenging. The current limitations of studying gut flora and its metabolites in BA should motivate scientists to elucidate newer approaches with modern technologies and explore more options regarding the interactions of gut microbiota, its metabolites, and BA in pathogenesis. The treatment of the gut microbiota and its metabolites in BA patients seems promising in the near future. Large-scale randomized controlled trials in patient are needed to evaluate the beneficial properties of probiotics, prebiotics, and synbiotics, their ideal dosages, duration of supplementation, persistence of their beneficial effects, and their safety in preventing and treating BA.

## Author contributions

SF: Investigation, Supervision, Writing – original draft, Writing – review & editing. YC: Conceptualization, Investigation, Software, Supervision, Visualization, Writing – original draft, Writing – review & editing. CS: Software, Supervision, Writing – review & editing. CY: Supervision, Validation, Writing – review & editing. YL: Conceptualization, Funding acquisition, Resources, Writing – review & editing.
